# Addressing Intimate Partner Violence and Power in Intimate Relationships in HIV Testing Services in Nairobi, Kenya

**DOI:** 10.1007/s10461-020-02801-9

**Published:** 2020-02-06

**Authors:** Nicole Haberland, Charity Ndwiga, Katharine McCarthy, Julie Pulerwitz, Rose Kosgei, Margaret Mak’anyengo, Amelia Peltz, Vincent J. Wong, Sam Kalibala

**Affiliations:** 1 Population Council, One Dag Hammarskjold Plaza, New York, NY 10017 USA; 2 Population Council, Nairobi, Kenya; 3 Population Council, Washington, DC USA; 4 Kenyatta National Hospital, Nairobi, Kenya; 5 Office of HIV/AIDS, United States Agency for International Development, Washington, DC USA

**Keywords:** HIV testing services, Intimate partner violence, Power in relationships

## Abstract

Intimate partner violence (IPV) undermines women’s uptake of HIV services and violates their human rights. In a two-arm randomized controlled trial we evaluated a short intervention that went a step beyond IPV screening to discuss violence and power with women receiving HIV testing services during antenatal care (ANC). The intervention included training and support for HIV counselors, a take-home card for clients, and an on-site IPV counselor. One third (35%) of women (N = 688) reported experiencing IPV in the past year; 6% were living with HIV. Among women experiencing IPV, program participants were more likely to disclose violence to their counselor than women receiving standard care (32% vs. 7%, p < 0.001). At second ANC visit, intervention group women were significantly more likely to report that talking with their counselor made a positive difference (aOR 2.9; 95% CI 1.8, 4.4; p < 0.001) and felt more confident in how they deserved to be treated (aOR 2.7; 95% CI 1.7, 4.4; p < 0.001). Exploratory analyses of intent to use ARVs to prevent mother-to-child transmission and actions to address violence were also encouraging.

**Electronic supplementary material:**

The online version of this article (10.1007/s10461-020-02801-9) contains supplementary material, which is available to authorized users.

## Introduction

Meeting HIV epidemic control goals and closing gaps in the HIV testing to treatment cascade entails more than expanded testing and antiretroviral treatment (ART) provision; it entails active engagement with the structural barriers to treatment initiation and adherence. Prominent among these structural barriers is intimate partner violence (IPV) which requires urgent attention not only in and of itself, but also because of its impact on other health and development indicators, including those related to HIV testing services (HTS). For example, a systematic review and meta-analysis found IPV to be associated with significantly lower ART use, lower ART adherence, and lower odds of viral load suppression among women [1]. Similar results were found in a recent scoping review [2]. A study in Zambia found that women who reported experiencing IPV had significantly reduced odds of adherence to medications to prevent mother-to-child transmission, including a reduced likelihood of adherence to drugs during pregnancy, adherence to drugs postpartum, and adherence to giving the infant prophylaxis [3].

The number of women affected by IPV is significant. The World Health Organization (WHO) has estimated that 30% of ever-partnered women globally have experienced physical and/or sexual IPV, calling IPV a public health problem of “epidemic proportions” [4]. In Nairobi, Kenya, the most recent Demographic and Health Survey (2014 DHS) found that about a third (35%) of ever-married women reported experiencing physical and/or sexual violence at the hands of their intimate partner in the past 12 months [5].

Thus, increasingly, the recommendation is to incorporate attention to IPV in HIV testing services so as to support women’s dignity and rights, and so that we do not lose women experiencing violence in the testing to treatment cascade [6, 7]. What to do and how to do it, however, remains a question. WHO guidelines for IPV, for example, discourage universal screening, but note that routine inquiry is warranted in antental care (ANC) settings and, though further research is needed, it may also be warranted in HTS [8]. Screening and referral interventions have been found to be acceptable and welcomed by women [9, 10], though the identification of women experiencing IPV is often low when compared to estimated IPV prevalence. For example, a recent systematic review of brief screening interventions in healthcare settings found a median disclosure prevalence of 8 percent [11] and a study in Kenya found the same prevalence in an IPV screening feasibility study [9]. Moreover, even among those women who disclose, many do not follow up on referrals. This has led some researchers [9–11] to recommend testing interventions that take a step beyond IPV screening and referral. Recommendations include providing basic psychosocial support for IPV and information at the time of screening, or equipping counselors to discuss gender power inequality in relationships more broadly, including IPV as a manifestation of that inequality.

These recommendations are bolstered by both theory and qualitative research. The theory of gender and power has been used in the HIV field to articulate how gender norms and power inequalities in relationships increase women’s HIV risk through multiple pathways, including norms of male toughness and acceptance of IPV perpetration, violence as a way to control women and maintain male dominance, notions of masculinity that endorse multiple partners and condomless sex for men, and notions of femininity that value women’s compliance and placing others’ needs above their own [12–14]. IPV and inequalities in relationships are thus mutually reinforcing and both decrease women’s ability to avoid HIV risk.

Qualitative research on what women who experience violence want from health professionals suggests that what providers say, and the nature of the interaction may be more important than whether a woman discloses violence or not [15–17]. A review of qualitative studies by Feder and colleagues found several recurring themes across 29 articles (25 studies) [15]. These themes included, for example, that women want providers to be nonjudgmental and to raise the issue of IPV in a compassionate and sensitive manner. It is important to confirm that the violence is unacceptable and not their fault. Women want to deal with the issue at their own pace and not be pressured to disclose to the provider. Learning about available resources regardless of whether they are ready to pursue them and feeling supported were other key themes. These findings suggest why interventions where providers ask screening questions such as, “Has your partner ever hit, choked, or physically hurt you?” and rely on the woman to disclose and follow-up on a referral, tend to have low rates of IPV disclosure and referral uptake. Not only does this leave women who disclose but are not ready to pursue a referral without support, it also leaves the women who either do not yet recognize that they are being abused, or who are not yet ready to disclose, without support or knowledge about resources available.

To respond to these evidence gaps and recommendations, we rigorously tested a simple pilot intervention that aimed to take a step beyond IPV screening and discuss violence and power with all women receiving HIV testing services. Our theory of change was that if all women were engaged with this basic information during HTS they would be more likely to feel comfortable disclosing violence, would receive important first-line support regardless of whether they followed up on a referral, and even if they chose not to disclose, would receive valuable information about their rights and where to go if and when they decide to seek help. The primary hypothesis was that IPV knowledge would be greater in the experimental group compared to the control group. Our secondary hypothesis was that women who received the intervention would be more likely to feel supported than women in the control group, and our exploratory hypotheses were that women in the experimental group, compared to the control group, would be more likely to do something about the violence and adhere to ART.

## Methods

### Setting

The study took place in Kenyatta National Hospital’s (KNH’s) ANC clinic in Nairobi, Kenya, from February 2015 to August 2015. KNH is a teaching hospital and the oldest and largest public referral hospital in the East Africa region, with KNH’s ANC clinic receiving upwards of 1000 clients per month. All ANC clients at KNH receive HTS as part of their first ANC visit of their current pregnancy. Women can choose to opt out of HIV testing. While IPV screening is supposed to be part of standard HTS counseling in the ANC clinic, it is inconsistently implemented.

Referrals for IPV are to KNH’s Gender-Based Violence Recovery Centre (GBV Centre), located within the KNH complex, about 5 min walk from the ANC clinic [18]. The GBV Centre provides comprehensive post-rape care that includes emergency care such as post-exposure prophylaxis (PEP); treatment for sexually transmitted infections (STIs); trauma counseling; and psychological support via individual counseling, support groups, and connecting women to social workers. The Centre also arranges referrals to safe houses if the client’s home environment is unsafe, to police, and to organizations that offer legal services.

### Standard HTS Care

For HIV-negative mothers, standard HTS counseling in the ANC clinic consists of discussing a birth plan, couple counseling if accompanied by a partner, information on danger signs during pregnancy, importance of attending scheduled ANC visits, maternal nutrition, STI risk reduction, postpartum family planning, hygiene and self-care, infant feeding, and male partner engagement in ANC/childcare. Women are also supposed to be screened for IPV and referred to the GBV Centre if they are experiencing violence. Mothers with HIV, in addition to the preceding, receive counseling on CD4 counts, ART, importance of adherence, and specialized care such as mother-to-mother groups and nutrition clinics.

### Intervention

The pilot intervention was implemented from March to July 2015 and included four main parts. The first component was a provider training. All providers in the ANC clinic participated in a 1-day off-site training on GBV. While providers had previous training at KNH in IPV screening and care, this training provided a refresher and an orientation to the intervention and study. The training included participatory activities—for example, myths and facts about IPV, and how relationship inequalities affect women’s well-being—and evidence on the impact of IPV and power inequalities in relationships on HIV prevention and care. The HTS counselors also received hands-on practice conducting the intervention. The 28 HTS counselors—25 females and 3 males—included lay counselors with between 2 and 10 years of experience, and nurses with between 4 and 20 years of experience.

The second intervention component included counseling aids for providers to use during the post-test counseling session. One was a small tri-fold card with key messages and resources regarding IPV, power, and women’s health. Cards developed by Futures Without Violence [19] for clinical settings in the U.S. were adapted by the study team to meet the objectives of this study and setting. The cards included a panel on facts, such as prevalence of IPV in Nairobi, and that being in a relationship characterized by IPV or low power for the woman increases her risk for HIV and STIs and can harm her baby’s health. Another panel posed reflective questions about the nature of the relationship—for example, is my partner’s communication honest and open? Who is mostly responsible for making decisions? Does my partner value my opinions and respect my choices? Is my partner respectful and kind to me? Relationship control and IPV were the focus of a third panel and asked about controlling behavior, physical and sexual abuse, and whether the woman was afraid to ask about condom use or tell her partner she has an STI or HIV. A highlighted panel included messages to remember such as, “You matter. You have a right to be free from violence,” “You are not to blame if you are experiencing any form of abuse,” and “There are people here who can help you.” Two additional panels covered possible things that a woman could do if she is being hurt or feels powerless, and provided contact information for four places to turn to for help in Nairobi: The GBV Centre at KNH, the Women’s Rights Awareness Programme (which provides safe shelter), Nairobi Women’s Hospital Gender Violence and Recovery Centre, and Centre for Rights Education and Awareness. HTS counselors gave the card to the woman as a resource to keep or share.

Counselors used a counseling script that elaborated the main messages on the card. The script guided counselors to introduce the topic of IPV by saying something like, “We’ve begun giving this card to all our ANC clients so they know how to get help for themselves if they need it, or so they can help a friend or family member who might need it.” For the card’s relationships panel, for example, the script read, “I am talking with all my clients about their relationships. Even if you are not in a relationship now, this is still important information for the future. I want you to think about these questions… Is your partner kind to you? Does he respect you and support you? If so, that is great. That makes it easier for you to protect your health and your children’s health.” Other key messages in the script included, “Remember, you have a right to be free from violence and you deserve to be treated with respect” and “Even if you don’t need any of this support now, relationships change—remember you can always come back to KNH for help.” The enhanced counseling took on average 6.5 min longer to implement than standard HTS (29 min vs. 23 min), the most common length (mode) of both enhanced and standard HTS sessions was 10 min.

Third, an IPV counselor from the GBV Centre was posted on-site in the ANC clinic to handle all IPV referrals immediately, with the intent of cutting down logistical and time barriers women face when following-up on referrals to clinics elsewhere in the KNH complex. To further facilitate access to the IPV counselor, a volunteer peer counselor accompanied referred women to the IPV counselor and/or GBV Centre.

Finally, HTS counselors involved in the study attended support group sessions facilitated by experienced counselor supervisors from KNH’s mental health unit. The purpose of the sessions was to offer a space for the counselors to debrief and process vicarious trauma, and to discuss questions and strategies about how to handle different situations.

### Study design

A randomized controlled trial assessed indicators on the hypothesized pathway of change—i.e., whether clients were screened for IPV, whether clients disclosed IPV, whether they received a referral, and whether they followed up on a referral—and intermediate outcomes—i.e., our primary outcome: IPV knowledge; and secondary outcome: whether clients felt supported. While the study was not designed to assess whether the intervention helped women address IPV or improve antiretroviral (ARV) adherence (the intervention’s longer term aims), we examined intent to adhere to ARVs to prevent mother-to-child transmission and actions taken to address IPV as exploratory outcomes.

All first-visit ANC clients during the study period (882) were approached and screened for eligibility. Out of 852 eligible women, 698 (82%) agreed to participate. Reasons given for declining to participate in the study were time concerns and competing obligations. Consenting participants were randomly assigned 1:1 to either intervention (IPV-HTS) or control (standard HTS) counseling.

To minimize bias that could be the result of counselors’ skills, experience, or personality, all counselors provided both the intervention and the standard counseling. While this increased the risk of spillover, the importance of adhering to the protocol was emphasized in training and in regular check-ins. In addition, the clients’ folders included standard HTS materials or IPV-HTS materials, depending on the ANC clients’ assignment to the intervention or control group. Because IPV screening is part of standard HTS counseling, if women in standard care disclosed IPV to their HTS counselor, the counselors explained the services available, i.e., IPV counseling in the ANC clinic and the GBV Centre; and referred her based on the women’s situation and needs.

Review of clients’ ANC card and HTS forms supplemented survey questionnaires administered to participants. Participants were interviewed immediately after receiving their ANC and HTS services, and then interviewed again approximately 1 month later at a subsequent ANC visit. The first-round interview was conducted after the intervention for ethical and research design reasons. Had we interviewed women before they received their HTS, and if a participant disclosed experiencing violence to the interviewer, the interviewer would, for ethical reasons, offer empathy and refer the woman for IPV counseling before she even saw an IPV-HTS or standard HTS counselor. As identifying women who are experiencing violence and referring them for IPV counseling are part of the intervention, such support and referrals by the interviewer would have pre-empted the intervention. Thus, both women in the intervention and control groups were interviewed post-HTS. In the post-HTS interviews, survey questions asked every participant about IPV, and if women responded that they had experienced IPV, the interviewer referred the woman for counseling. The interviewers did this regardless of which study arm the woman was in.

### Inclusion/Exclusion Criteria

Inclusion criteria were: first-time ANC client (i.e., a woman presenting for her first ANC visit of her current pregnancy) and being aged between 15 and 49 years. Exclusion criteria were having received an HIV test in the previous 6 months.

### Data collection

Following their post-test HIV counseling, clients were interviewed by research staff using a structured questionnaire. Surveys were translated into Swahili and scales’ internal consistency (reliability) was assessed after adaptation to the local language. Questions covered socio-demographic information, clients’ relationship status, partner characteristics, condom use, HIV testing, couples HTS, disclosure of HIV status, knowledge of partner’s status, and perceived difficulty disclosing or asking about a partner’s HIV status. Power inequalities in the relationship were assessed using the Sexual Relationship Power Scale (SRPS) (Cronbach’s α = 0.67) [12]. As recommended, the Relationship Control and Decision-making Dominance subscales of the overall SRPS were combined and rescaled to have a final score ranging from 1 to 4, with a higher score indicating greater relationship power for the woman. Attitudes toward gender norms were measured using a South African adaptation of the GEM Scale (Cronbach’s α = 0.80) [20, 21]. The total scale items were averaged so that the overall scale ranged from 1 to 3, with a higher score indicating greater endorsement of gender equitable norms.

We asked about women’s experience of emotional, physical, and sexual violence in her lifetime, in the past 12 months, and by current and other partner/s. For example, for emotional violence, women were asked whether in the past 12 months her current partner had insulted her or made her feel bad about herself; belittled or humiliated her in front of other people; did things to scare or intimidate her on purpose; or threatened to hurt her or someone she cared about. If the answer to each of these questions was ‘no,’ they were followed up with whether her current partner had ever done any of these things; whether another partner had done these things in the past 12 months, or if anyone had ever done these things. We also asked whether the woman had ever sought help for IPV. For most analyses, we operationalized IPV as any IPV—emotional, physical, or sexual—experienced in the past 12 months. Where noted, we also looked separately at those women who reported physical and/or sexual violence only in the past 12 months. Finally, the questionnaire also asked women about their HTS counseling experience, including whether she disclosed violence to the counselor and her follow-up or intent to follow-up on the referral. Information on HIV status was extracted from participants’ ANC medical charts.

A second interview was conducted with participants at a subsequent ANC visit (mean duration between interviews was 4.4 weeks) to ascertain whether women had followed up on referrals, assess whether any beneficial effects were sustained, and to monitor whether she had experienced any adverse outcomes due to the intervention. We asked relevant behavioral and self-efficacy questions, such as confidence in ability to regularly take ARVs to prevent maternal to child transmission, to explore whether the intervention had the potential to improve these outcomes.

### Analysis

Sample size was calculated to detect a 13% point difference between study arms in IPV-related knowledge and awareness. As no prior data on these indicators existed, 57% baseline prevalence was assumed using endorsement of IPV from the DHS as a proxy variable [22]. Calculations were based on the following additional assumptions: β = 0.80, α = 0.05, 10% non-response rate, and 25% attrition while applying the continuity correction. The study was not powered to assess changes in subgroup analyses (e.g., among women living with or without HIV).

Statistical analysis was performed in R Studio Version 3.1 (R Studio Inc., Boston, MA), using an intent-to-treat approach. Chi-square and two-sample t-tests were used to assess whether women systematically differed on sociodemographic characteristics, endorsement of gender norms, or relationship characteristics with regard to study arm or attrition status. Bivariate analyses were also used to assess cross-sectional differences between intervention and control group participants at first and second follow-up. Pairwise missing data were excluded from analysis. We used multivariate logistic and linear regression analysis to assess differences in outcomes at each follow-up time by intervention assignment. Differences were considered statistically significant at p < 0.05.

### Ethical Review

The study protocol was reviewed and approved by the Population Council Institutional Review Board (New York), and the Kenyatta National Hospital/University of Nairobi Ethics and Research Committee. Written informed consent was obtained from each respondent prior to study participation.

### Trial Registration

ClinicalTrials.gov #NCT02577380.

## Results

A total of 698 ANC clients attending KNH’s ANC clinic during the enrollment period met study eligibility criteria, provided written informed consent, and were randomized to intervention or control groups. Ten questionnaires had conflicting data on intervention assignment that could not be resolved and were excluded from analysis. We successfully interviewed 535 of 688 women at a next ANC visit (78% retention). Of the women lost to follow-up, most did not return to KNH for a subsequent ANC visit because they had delivered their baby, changed clinics, or did not pursue additional ANC visits. Study arms did not differ with respect to attrition rate. There were no significant differences in the types of respondents who remained in the study and those lost to follow-up in terms of age, education, marital status, HIV status, or experience of IPV.

### Participant Characteristics

Women participating in the study were, on average, 29 years old (Table 1). Half (49.6%) of participants had completed some tertiary education. Four-fifths (82%) were currently married; of the remainder (data not shown) 2% were unmarried but living with a man, 9% had a regular partner but were not living together, and 6% were single. Based on HIV data in ANC client charts, 5.7% of women were living with HIV. Approximately 8% of women reported food insecurity. Women’s average scores were 2.5 on the GEM Scale (with 1 being the least and 3 being the most endorsement of equitable gender norms) and 2.5 on the SRPS (with 1 being a low power position for women and 4 being a high power position). Thirty-eight percent of women reported ever experiencing IPV.

**Table 1:** Sample characteristics by study arm

	Total N = 688 (%)	Intervention N = 337 (%)	Control N = 351 (%)	P-value^a^
Age group				
15–19	1.6	1.1	2.1	0.31
20–24	16.4	16.9	15.9	
25–29	34.1	35.0	33.2	
30–34	29.9	31.2	28.4	
35–39	15.4	12.6	18.3	
40–45	2.6	3.2	2.1	
Mean age (SD)	29.4 (± 5.2)	29.2 (± 5.2)	29.6 (± 5.3)	0.26
Education				
Primary	17.8	19.1	16.4	0.47
Secondary	32.6	33.4	31.6	
College/University	49.6	47.4	51.9	
Marital status^b^				
No	18.1	16.6	19.7	0.32
Yes	81.9	83.4	80.3	
HIV status				
Negative	94.3	94.5	94.1	0.86
Positive	5.7	5.5	5.9	
Food insecure				
No	91.9	92.3	91.4	0.68
Yes	8.1	7.7	8.6	
Gender equitable norms^c^ Mean (SD)	2.5 (± 0.4)	2.4 (± 0.4)	2.5 (± 0.4)	0.08
Sexual relationship power^d^ Mean (SD)	2.5 (± 0.4)	2.5 (± 0.4)	2.5 (± 0.4)	0.009
Ever experienced IPV				
No	62.1	59.2	65.2	0.11
Yes	37.9	40.8	34.8	
IPV in past 12 months (any type)				
No	65.0	61.8	68.2	0.008
Yes	35.0	38.2	31.8	
Emotional IPV in past 12 months				
No	70.9	68.1	73.9	0.11
Yes	29.1	31.9	26.1	
Physical IPV in past 12 months				
No	85.8	85.1	86.5	0.66
Yes	14.2	14.9	13.5	
Sexual IPV in past 12 months				
No	87.1	85.6	88.6	0.26
Yes	12.9	14.4	11.4	
Physical or sexual IPV in past 12 months				
No	78.7	77.3	80.2	0.40
Yes	21.3	22.7	19.8	
Days between survey rounds				
Mean (SD)	30.9 (± 20.4)	31.0 (± 20.4)	30.9 (± 20.5)	0.99

^a^Chi-square test for categorical variables and independent t-test for continuous variables. Significance at p < 0.05

^b^Married or living together

^c^GEM scale measured on 1 to 3-point scale with higher score indicating more equitable views on gender norms

^d^SRPS measured on 1 to 4-point scale with higher score indicating more equitable relationships

Of the women who had ever experienced IPV, the vast majority—93% (241 out of 258)—had experienced it in the past year. Emotional IPV was reported by 29% of women; physical IPV was reported by 14% of women; and sexual violence was reported by 13% of women. One in five (21%) of women reported experiencing physical or sexual violence. The intervention and control groups do not differ statistically on any type of reported IPV, although “any IPV in past 12 months” approached significance (p = 0.079).

In a supplemental table we show differences in HIV status and HIV disclosure behaviors by experience of IPV in the past 12 months. HIV prevalence was significantly higher among women who experienced physical and/or sexual violence in the past year relative to women who did not experience either form of violence (10% vs. 4%, p = 0.018).

### Intervention Effects

Women in the intervention group were far more likely to report IPV screening than women in standard care (86% vs. 21%, p < 0.001, data not shown). Among women who in the survey reported experiencing any IPV in the past year, the same difference is evident: 76% of IPV-positive women in the intervention reported being screened for violence compared to 22% of IPV-positive women in the control group (p < 0.001; Table 2). IPV-positive women in the intervention group were also significantly more likely to disclose that they were experiencing IPV than IPV-positive women in the control group (32% vs. 7%, p < 0.001). Among women who had experienced violence in the past year, those in the intervention group were twice as likely to report following-up on referrals compared to women in the control group, though the difference was not significant (15% vs. 8%, p = 0.164).

**Table 2:** Reports of IPV screening, disclosure of IPV, and follow-up by women who experienced any IPV in the past 12 months

	Women reporting any violence in past 12 months^a^			
	Total N = 241 (%)	Control N = 134 (%)	Intervention N = 107 (%)	P-value^b^
Screened for IPV (Follow-up 1)				
No screening	54.2	78.2	24.3	< 0.001
Provider screened	45.8	21.8	75.7	
Disclosed IPV (Follow-up 1)				
No	82.2	93.3	68.2	< 0.001
Yes	17.8	6.7	31.8	
Followed-up on referral^c^ (Follow-up 2)				
No	89.0	92.2	85.2	0.164
Followed-up/intend to today	11.0	7.8	14.8	

^a^Including physical, sexual or emotional

^b^Chi-square tests for association. Significance at p < 0.05

^c^Total response on variable N = 191 (103 control, 88 intervention) due to attrition at second follow-up and one case of missing data

Participants’ knowledge of women’s rights vis a vis IPV was high. For example, over 93% of participants were aware that women can legally divorce a husband due to cruel treatment, irrespective of intervention group or time of follow up. IPV knowledge items (ranging from 0 to 6) were summed, showing a small effect of the intervention which was marginally significant at second follow-up after adjusting for possible covariates (Adj. Beta 0.155; 95% CI − 0.01, 0.31; p = 0.051; Table 3).

**Table 3:** IPV-related knowledge by study arm at second follow-up

Mean comparison			Unadjusted linear regression model		Adjusted linear regression model^a^	
Control N = 267 Mean (95% CI)	Intervention N = 268 Mean (95% CI)	P-value	Intervention (vs. control) Βeta (95% CI)	P-value	Intervention (vs. control) Adj. Βeta (95% CI)	P-value
3.14 (3.03, 3.25)	3.31 (3.20, 3.43)	0.014*	0.176* (0.02, 0.33)	0.028*	0.155 (− 0.01, 0.31)	0.051

^a^Covariates include IPV experienced in the past 12 months and sexual relationship power

^*^p < 0.05

Women’s reports of the support they received in their HTS session are shown in Table 4. Women in the intervention group were significantly more likely to report better results across all indicators and at both follow-up points, with the exception of “feeling better able to take care of their health” in which the difference was no longer significant at second follow-up. In the multivariate analysis, women in the intervention group were significantly more likely than women in the control group to report at second follow-up that talking with their counselor made a positive difference (aOR 2.9; 95% CI 1.8, 4.4; p < 0.001), they learned new things about a woman’s rights in her relationship (aOR 3.7; 95% CI 2.3, 6.1; p < 0.001), and felt more confident in how they deserved to be treated (aOR 2.7; 95% CI 1.7, 4.4; p < 0.001).

**Table 4:** Regression model of perceived intervention support, by study arm at first and second follow-up using logistic regression analysis

	Unadjusted intervention (vs. control)		Adjusted^a^ intervention (vs. control)	
	OR (95% CI)	P-value	aOR (95% CI)	P-value
Talking with provider made positive difference^b^				
Follow-up 1				
Agree a lot (vs. agree somewhat or disagree)	2.83 (1.78, 4.52)	< 0.001	2.76 (1.72, 4.42)	< 0.001
Follow-up 2				
Agree a lot (vs. agree somewhat or disagree)	2.95(1.89, 4.60)	< 0.001	2.89 (1.84, 4.41)	< 0.001
Learned new things about a woman’s rights in her relationship^b^				
Follow-up 2^c^				
Agree a lot (vs. agree somewhat or disagree)	3.79 (2.32, 6.19)	< 0.001	3.72 (2.27, 6.10)	< 0.001
Feel better able to take care of health than before visit^d^				
Follow-up 1				
Better (vs. same)	3.83 (2.03, 7.22)	< 0.001	4.00 (2.08, 7.71)	< 0.001
Follow-up 2				
Better (vs. same)	1.23 (0.50, 3.03)	0.643	1.15 (0.47, 2.85)	0.754
Feel more confident in how deserve to be treated^d^				
Follow-up 1				
Better (vs. same)	4.96 (3.30, 7.44)	< 0.001	5.03 (3.33, 7.86)	< 0.001
Follow-up 2				
Better (vs. same)	2.81 (1.76, 4.49)	< 0.001	2.72 (1.70, 4.36)	< 0.001

^a^Adjusted analyses also included sexual relationship power and experience of intimate partner violence within the past 12 months

^b^Response dichotomized as ‘Yes a lot’ vs. ‘A little’, ‘A fair amount’ or ‘Not at all’

^c^Variable not comparable between rounds and only Follow-up 2 is displayed

^d^Response coded as ‘Better’, ‘Same’ or ‘Worse’. Note: No women responded ‘Worse’

Significance at p < 0.05

While our sample was not powered to detect changes among subgroups, we conducted exploratory analyses to assess whether there were differences among women who reported experiencing any IPV in the past 12 months (Table 5). At second follow-up, agency for asking partner to go to couples HTS tended to be higher in the intervention group than the control group (though not significantly so) (76% vs. 71%, p = 0.59), and so were: being better able to take care of one’s health/well-being (88% vs. 80%, p = 0.16), asking partner to use a condom (58% vs 49%, p = 0.31), and intending to regularly take medications to prevent mother-to-child transmission (among those women living with HIV, 62% vs. 48%, p = 0.38).

**Table 5:** Exploratory analysis: agency and health-promoting behaviors among women who reported experiencing IPV in the past 12 months, by study arm at second follow-up

	Women reporting any violence in past 12 months^a^			
	Total (%) N = 241	Control (%) N = 134	Intervention (%) N = 107	P-value^b^
Could ask partner to go to couples HTC (CHTC)				
Agree a lot	73.2	71.3	75.7	0.59
Agree somewhat or disagree	26.8	28.7	24.3	
My partner and I have discussed going to CHTC				
Agree a lot	55.2	55.7	54.7	1.00
Agree somewhat or disagree	44.8	44.3	45.3	
Can take better care of my health/well-being				
Agree a lot	83.9	80.2	88.2	0.16
Agree somewhat or disagree	16.1	19.8	11.8	
I will regularly take medications to prevent mother-to-child transmission^c^				
Agree a lot	54.5	47.8	61.9	0.38
Agree somewhat or disagree	45.5	52.2	38.1	
I can ask my partner to use a condom				
Agree a lot	52.8	48.8	58.3	0.31
Agree somewhat or disagree	47.2	51.2	41.7	

^a^Including physical, sexual or emotional; all indicators initially assessed on 5-point scale: Agree a lot, Agree somewhat, Neither agree nor disagree, Disagree somewhat, Disagree a lot; then dichotomized as indicated

^b^Chi-square tests for association. Significance at p < 0.05

^c^Among those women living with HIV

Finally, we conducted exploratory analyses of IPV-related outcomes (Table 6). Among women who had experienced any type of violence in the past year, a higher proportion in the intervention group reported taking any action to address the violence (i.e., following up on a referral, leaving a partner, telling someone, or going somewhere for help) compared to control (36% vs. 26%, p = 0.09) at second follow-up. Among those women experiencing physical or sexual violence in the past year, the proportion of women taking any action was also higher in the intervention group compared to the control (46% vs. 31%, p = 0.07).

**Table 6:** Exploratory analysis: women’s actions since first follow-up interview among those experiencing IPV in the past 12 months, by study arm at second follow-up

	Total (%)	Control (%)	Intervention (%)	P-value^a^
Women reporting any violence in past 12 months^b^	N = 241	N = 134	N = 107	
Followed up on referral				
No	89.0	92.2	85.2	0.095
Yes	11.0	7.8	14.8	
Left partner				
No	66.1	73.5	57.1	0.191
Yes	33.9	26.5	42.9	
Told someone				
No	83.9	85.4	82.0	0.559
Yes	16.2	14.6	18.0	
Went anywhere for help				
No	91.2	94.1	88.2	1.00
Yes	8.8	5.9	11.8	
Any of above^c^				
No	69.1	73.8	63.6	0.088
Yes	30.9	26.2	36.4	
Women experiencing physical and/or sexual violence in past 12 months	N = 145	N = 79	N = 66	
Followed up on referral				
No	86.0	89.8	81.8	0.163
Yes	14.0	10.2	18.2	
Left partner				
No	57.5	70.0	45.0	0.100
Yes	42.5	30.0	55.0	
Told someone				
No	79.1	81.4	76.8	0.354
Yes	20.9	18.6	23.2	
Went anywhere for help				
No	88.5	91.7	85.7	1.00
Yes	11.5	8.3	14.3	
Any of above^c^				
No	62.3	69.5	54.6	0.073
Yes	37.7	30.5	45.5	

^a^Chi-square tests for association. Significance at p < 0.05

^b^Including physical, sexual or emotional

^c^Yes to any of following categories: followed up on referral, left partner, told someone, or went anywhere for help

## Discussion

IPV is widely recognized as a pandemic that must be addressed as a problem in its own right, and because of its deleterious effects on health and development, including on outcomes along the HIV prevention, treatment and care continuum. Because IPV may affect disclosure of HIV status, ability to follow-up on treatment, and the safety of women who disclose, HTS has been identified as a service delivery point that can contribute to a multi-sector response to IPV, though further research is needed [8]. We tested a pilot intervention that sought to provide all ANC clients receiving HTS, regardless of whether they screened positive for IPV, with basic information and resources about IPV and power inequalities in relationships during post-test HIV counseling.

Although women who received IPV/power-enhanced counseling had greater IPV knowledge compared to women in the control group, after adjusting for possible covariates the difference was just marginally significant at p = 0.051, possibly because of relatively high knowledge levels among all women in the study. IPV/power-enhanced counseling significantly increased the proportion of IPV-positive women who disclosed IPV to their HTS counselor compared to standard care. The proportion of women in the intervention who followed up on referrals for IPV services was double that among women in the control group, though this difference was not significant, likely because of the small sample size. The percent of women who perceived meaningful support from the HTS session was significantly higher among women in the intervention than in the control group, a difference that was maintained after controlling for other variables and at second follow-up. This perception of support may be an important intermediate outcome as other studies have found that the provision of social support to women experiencing IPV is linked with improved physical and mental health outcomes [23, 24]. Finally, per responses to the survey, recent IPV affected a full third of the ANC clients, yet only a third of women experiencing IPV in the intervention arm—and a much smaller percent in the control arm—disclosed to their counselor. Thus, in many instances the HTS counselors did not know that their client was experiencing violence. In the intervention arm, however, because counselors discussed IPV with all clients, women who chose not to disclose IPV to their HTS counselor still received supportive messages and important information about IPV and resources available in Nairobi.

While this study was not powered to assess effects among HIV-positive and IPV-positive subgroups on indicators related to HIV care and prevention, or IPV prevention and treatment, exploratory analyses are encouraging. Of note are the higher proportions of IPV-positive women reporting intention to regularly take ARVs to prevent mother-to-child transmission and agency to ask their partner to use a condom (not significant). Among women experiencing physical or sexual violence, the percentage of women who took some sort of action to address the violence was higher (marginally significant) in the intervention than the control group (46% vs. 31%, p = 0.07).

Based on these results, providing brief IPV/power counseling to all women—not just screening, and not just to those who disclose violence—may be warranted in settings such as HTS and ANC and should be tested with a larger sample and over a longer period of time in order to track outcomes such as ARV adherence and women’s experience of IPV. Counselors’ feedback suggests that the provider support groups are an important feature of the intervention to maintain, as are the escorts who brought women to their referral destination.

We note several limitations and considerations. The findings, while encouraging, and obtained with a rigorous randomized design, have limited generalizability. Our study population was urban, and more highly educated than the general Kenyan population. Approximately half of study participants reported more than secondary education, compared to a quarter (24%) of women in Nairobi reporting this in the DHS [5]. Also, we note that KNH is an unusual hospital in terms of the pre-existing awareness and infrastructure for addressing IPV. Replicating this intervention in other settings would entail more upfront awareness raising and likely more work to establish referral linkages. While we sought to ensure that no spillover occurred between intervention and control groups, it is possible that some counselors, intentionally or not, at times provided all or part of the intervention to women in the control group. This could have led to an underestimation of the effect of the intervention. Additionally, due to ethical reasons, it was not possible to conduct a baseline assessment, as women who reported IPV in the survey would warrant counseling and referral for services. It was therefore not possible to account for baseline levels of variables although randomization should have ensured equivalence between groups.

The study illuminated some important questions for future evaluations. One is whether it would be better to randomize at the cluster (health facility) level as opposed to the individual level. While this would help avoid spillover, it too carries drawbacks as health facilities differ from one another on a host of characteristics that could bias results. While randomization should take care of that to some degree, it does not always do so in practice. Second, the fact that a substantial proportion of women who did not disclose IPV to their HTS counselor but subsequently acknowledged experiencing violence during the survey is interesting. A possible explanation is that the IPV counseling during the HTS session prepared the woman and even though she was not ready to disclose during HTS, she had a little time to process it and thus felt more comfortable disclosing IPV when asked in the interview. Indeed, data from Feder and colleagues shows that women tend to want to deal with the issue at their own pace [15]. Research to determine why this happened could provide useful insight for future research and interventions. For example, if asking women about their IPV experience multiple times facilitates disclosure, discussing IPV could be built into all of a woman’s ANC visits, not just her first ANC visit for a pregnancy.

The pilot intervention—using simple tools and counselor training and support—demonstrated some significant positive intermediate effects, as well as encouraging exploratory results, despite adding only 6.5 min to HTS counseling time. Six and a half minutes is not a small amount of time in high-volume clinics, indeed providers at KNH expressed concern about the added time given their client load. Nonetheless, they all still viewed the intervention as worthwhile and in many HTS clinic settings such an intervention may be easily scalable. While UNAIDS reports progress towards achieving the 90–90–90 treatment targets, stretching the final mile will entail enhancing clinical services to address structural factors such as IPV that hinder HIV treatment initiation and adherence. Knowing whether a woman is experiencing IPV can allow for better tailoring of the HIV counseling session to her situation—including how best to immediately start and stay on ART should she fear her partner’s reaction—but it also offers the opportunity to provide immediate support to her. Such immediate, “first-line” assistance for IPV includes being non-judgmental and supportive; listening carefully and empathetically; and helping her access information and legal, social, and health resources, including, if indicated, trauma counseling and post-exposure prophylaxis [8]. Findings from this study suggest—though the study was not powered to determine—that in addition to providing more appropriate care, there are potentially positive health and behavioral benefits to including IPV/power in HTS counseling. A larger study, incorporating lessons from this pilot is warranted.

## Electronic supplementary material

Below is the link to the electronic supplementary material.

Supplementary material 1 (DOCX 17 kb)
