# Supplementary material for: Addressing Intimate Partner Violence and Power in Intimate Relationships in HIV Testing Services in Nairobi, Kenya
Source: AIDS Behav. 2020 Feb 6;24(8):2409–20. doi: 10.1007/s10461-020-02801-9 (PMC7395047; doi:10.1007/s10461-020-02801-9)
Supplement: Supplementary file 1 — Supplementary material 1 (DOCX 17 kb) [file 10461_2020_2801_MOESM1_ESM.docx]

**Supplemental Table. HIV Disclosure Behaviors and Status among Women**

**by Experience of IPV in the Past 12 Months Reported at Follow-up 1**

|  | **Total**  (%) | **IPV Negative**  (%) | **IPV Positive**  (%) | **P-value^a^** |
| --- | --- | --- | --- | --- |
| **Women Experiencing Any Violence in Past 12 Months** | N=688 | N=455 | N=243 |  |
| **Disclosed HIV status to partner** |  |  |  |  |
| No | 13.5 | 10.6 | 18.6 | 0.004 |
| Yes (positive or negative result) | 87 | 89 | 81 |  |
| **Know partner’s HIV status** |  |  |  |  |
| No | 23.3 | 21.1 | 27.4 | 0.072 |
| Yes (positive or negative result) | 76.7 | 78.9 | 72.6 |  |
| **Have ever been to couples HTC**^b^ |  |  |  |  |
| No | 33.3 | 29.2 | 40.8 | 0.002 |
| Yes | 66.7 | 70.8 | 59.2 |  |
| **HIV status** |  |  |  |  |
| Negative | 94.3 | 95.4 | 92.3 | 0.140 |
| Positive | 5.7 | 4.6 | 7.7 |  |
| **Women Experiencing Physical and/or Sexual Violence in Past 12 Months** | N=688 | N=543 | N=145 |  |
| **Disclosed HIV status to partner** |  |  |  |  |
| No | 13.5 | 11.2 | 22.0 |  |
| Yes (positive or negative result) | 86.5 | 88.8 | 78.0 | 0.001 |
| **Know partner’s HIV status** |  |  |  |  |
| No | 23.4 | 21.5 | 30.3 |  |
| Yes (positive or negative result) | 76.6 | 78.5 | 69.7 | 0.026 |
| **Have ever been to couples HTC**^b^ |  |  |  |  |
| No | 33.4 | 31.3 | 41.0 |  |
| Yes | 66.6 | 68.7 | 59.0 | 0.030 |
| **HIV status** |  |  |  |  |
| Negative | 94.4 | 95.6 | 90.0 | 0.018 |
| Positive | 5.6 | 4.4 | 10.0 |  |

^a^ Chi-square tests for association. Significance at p<0.05.

^b^ HTC refers to HIV testing and counseling.
